# A Polypeptide-DNA Hybrid with Selective Linking Capability Applied to Single Molecule Nano-Mechanical Measurements Using Optical Tweezers

**DOI:** 10.1371/journal.pone.0054440

**Published:** 2013-01-15

**Authors:** Fatemeh Moayed, Alireza Mashaghi, Sander J. Tans

**Affiliations:** 1 AMOLF Institute, Amsterdam, The Netherlands; 2 Department of Bionanoscience, Kavli Institute of Nanoscience, Faculty of Applied Sciences, Delft University of Technology, Delft, The Netherlands; Consejo Superior de Investigaciones Cientificas, Spain

## Abstract

Many applications in biosensing, biomaterial engineering and single molecule biophysics require multiple non-covalent linkages between DNA, protein molecules, and surfaces that are specific yet strong. Here, we present a novel method to join proteins and dsDNA molecule at their ends, in an efficient, rapid and specific manner, based on the recently developed linkage between the protein StrepTactin (STN) and the peptide StrepTag II (ST). We introduce a two-step approach, in which we first construct a hybrid between DNA and a tandem of two STs peptides (tST). In a second step, this hybrid is linked to polystyrene bead surfaces and Maltose Binding Protein (MBP) using STN. Furthermore, we show the STN-tST linkage is more stable against forces applied by optical tweezers than the commonly used biotin-Streptavidin (STV) linkage. It can be used in conjunction with Neutravidin (NTV)-biotin linkages to form DNA tethers that can sustain applied forces above 65 pN for tens of minutes in a quarter of the cases. The method is general and can be applied to construct other surface-DNA and protein-DNA hybrids. The reversibility, high mechanical stability and specificity provided by this linking procedure make it highly suitable for single molecule mechanical studies, as well as biosensing and lab on chip applications.

## Introduction

Many experiments involving the manipulation of nucleic acids and proteins require multiple strong linkages that can be established *in-situ*, and can be used together and thus must be specific. For certain applications the molecules involved are immobilized on surfaces, either because the experimental setup requires fixing and controlling the position of the molecular ends or because the molecular phenomenon is measured using surface sensitive techniques [1], [2]. An example of an experiment demanding such supramolecular structures at surfaces includes the binding of liposome-ssDNA hybrids to surface immobilized-DNA in order to detect single nucleotide polymorphism usingtotal internal reflection fluorescence (TIRF) microscopy [3]. Another example is the large-scale positioning of self-assembled functional DNA nanoarrays on surfaces [4], which have been used to construct arrays of quantum dots, proteins, and DNA targets. Supramolecular constructs that link micron-sized beads have been used to engineer molecular wires and to guide the assembly of nano and microstructures [5]–[8]. Metal wires have been fabricated by depositing metals on multi-protein and DNA constructs connecting the surfaces of two electrodes [9], [10].

Single molecule techniques such as optical tweezers have enabled the kinetic and thermodynamic characterization of DNA and protein molecules, as well as their interaction [11]–[14]. In these methods, two ends of the molecule of interest typically are manipulated by linking them to surfaces, either directly or via molecular handles. Here molecular linkages are preferably established *in-situ* while still being able to sustain large forces over long timescales. Different classes of linkages have been used: Antibody-antigen linkages [13], the family of Streptavidin (STV)-biotin linkages [13]–[15], covalent disulfide linkages [14] and covalent binding proteins (HaloTag [16] or SNAP-tag [17]). Each has its own strength and drawbacks. Antibody-antigen interactions are specific and diverse but affinities are affected by buffer condition, pH and temperature, and thus limit the experimental conditions that can be explored. Examples are Myc-AntiMyc and Dig-AntiDig. Moreover, many commercially available antibodies are polyclonal, causing variability in the force that the linkage can sustain. The Dig-AntiDig connection can be stable mechanically, and has therefore been used extensively to link DNA to surfaces [13], [14]. However, this system is less suitable for interfacing to proteins, while as a steroid compound [18]. Digoxigenin is also prone to oxidation and thus can deteriorate over time [19]. Disulfide bonds are very strong but involve long preparation times (e.g. 24–48 hr for DNA-protein coupling [20]) and the molecules of interest must be resistant to redox reactions, which limits its applicability.

The biotin-STV interaction is one of the most broadly used, as it is strong and efficiently established. STV is one of the most stable proteins showing high resistance to temperature, urea, guanidine, and proteases [21]. This is in contrast to linkages such as HaloTag or SNAP-tag that unfold, aggregate and encourage nonspecific binding under these harsh conditions [15]. In the presence of SDS, Streptavidin begins to break up into monomers only at temperatures above 60°C [22]. Because of the usefulness of biotin-STV interactions, efforts have been made to engineer variants and further optimize this system. Avidin is a glycosylated and positively charged protein (at neutral pH) which usually appears as a tetrameric biotin-binding molecule. Neutravidin (NTV) is a deglycosylated form of Avidin which is developed to decrease non-specific interactions [23]. It has recently been reported that Traptavidin, a mutant of STV, dissociates biotin more than tenfold slower, has increased mechanical strength and improved thermostability [15].

StrepTactin (STN) is another, recently engineered version of STV which has high affinity to biotin and in particular to its peptide ligand (K_d_≈1 μM), named StrepTag II (ST), which is 8 amino acids long (WSHPQFEK) [24]. STN has a tetrameric structure that provides four binding sites for ST. Additionally the binding can be reversed by adding Desthiobiotin which can in turn be removed by washing or dialysis. This feature has made the system popular for the purification and detection of proteins by affinity chromatography [24]. Interestingly, STN does have affinity for biotin [24] and ST can bind STV (Kd ≈72 μM) at the same surface pocket where biotin is complexed [25] while ST cannot bind Avidin (AV) [25], [26]. Because the biotin binding pockets in NTV and AV have similar surface structures, one may expect that NTV – like AV – is unable to bind ST. It has been reported that the binding affinity of ST to STV can be further increased to nanomolar levels when using multiple tandem STs [27]. It is also shown that in protein purification, having multiple tandem STs improves the binding affinity to STN [24]. ST can be cleaved enzymatically, and the ST-STN interaction is resistant to reducing agents (DTT and mercaptoethanol), denaturing agents (urea 1 M), chelating agents (EDTA 50 mM) and detergents (SDS 0.1% and Triton X100 2%). ST is proteolytically stable, biologically inert and does not interfere with membrane translocation or protein folding [24]. The strength of the STN-ST linkage has been recently studied by Atomic Force Microscopy [28], [29], in which one single ST was fused to a protein and STN was anchored to a surface via PEG-based [29] or long protein-based [28] handles. The linkage showed an average dissociation force of 40 and 60 pN at pulling rates of 337 and 200 nms^−1^, respectively [28], [29]. It is unclear what the dissociation force is for STN that is immobilized directly on the surface, and for multiple ST binding to a single STN.

The properties of the ST-STN linkage show promise for use in optical tweezers experiments and biomaterial engineering. These applications typically require multiple linkages that are specific and strong, which ST-STN can potentially deliver. One challenge is to construct polypeptide-DNA hybrids, which would be required for such an approach. Oligonucleotides (6–16 mers) conjugated to a tripeptide have been used for PCR amplification to successfully construct hybrids of DNA with short polypeptides [30]. The feasibility of synthesizing oligonucleotides conjugated to long polypeptides, and using them to amplify DNA segments, remains unclear.

We present a straightforward method to efficiently construct end-joined molecular hybrids in a manner that is mechanically stable and specific. To increase the stability [24], [27], our method uses a tandem two STs (tST)-STN linkage to couple two molecules A and B, where both A and B can potentially be either DNA or protein of arbitrary size. Here we demonstrate the coupling of Maltose Binding Protein to a 920 nm long dsDNA. We find that DNA molecules can be coupled well to the surface via tST-STN linkage. The linkage is more stable against applied force than the biotin-STV linkage and can be used in conjunction with biotin-NTV to stably tether DNA and to construct protein-DNA hybrids.

## Materials and Methods

### Design and synthesis of the oligo-peptides

A tandem arrangement of two STs (tST: WSHPQFEKWSHPQFEK) was chemically synthesized and was linked to the primer (5′GTC TCG CGC GTT TCG GTG ATG ACG GTG 3′) from its 5′ end via a linker (-Cys-SMCC-C6) (BioSynthesis Inc.). The product was purified by HPLC and characterized by mass spectrometry (Applied Biosystems Voyager System 2051).

### Synthesis of dsDNA-tST

The 2553 bps DNA handles were generated by PCR using Taq DNA polymerase and pUC19 plasmid DNA (New England BioLabs) as template. 500 ng of handles were generated at a time using 50 μl of PCR reaction. The two types of handles (with and without biotin) were generated using the above oligo-peptide as a forward primer together with the primer 5′ TA6GTA6CCGCTCATGAGAC 3′ as a reverse (6 is biotin-dT for biotinylated DNA and is “T” for non-biotinylated DNA). Polymerase chain reaction reagents for each 50 microliter reaction volume included: 1 unit of Taq polymerase (New England BioLabs), 5 μl of 10x PCR buffer (New England BioLabs), 10 pmol of the forward primer and 10 pmol of reverse primer, 5 μl of 2 mM dNTPs (Fermentas), and 50 ng of the plasmid DNA. The PCR profile was as follows: 1 min at 94°C, 30 cycles of 30 s at 94°C, 60 s at 52°C and 3 min at 72°C, finally followed by 10 min at 72°C and a 4°C soak.

### Expression and purification of Maltose Binding Protein (MBP)

Two repeats of the sequence encoding the ST (tST) were introduced with PCR at the C-terminus of MBP sequence using plasmid pNN226 as a template. The inserts were ligated with HindIII/NdeI restriction sites to the pET3 vector to generate the expression plasmid. The correctness of the newly made vector was confirmed by double-strand DNA sequencing. *Escherichia coli* strain BL21.1 was used to express the MBP construct. The cells were grown at 37°C in LB medium containing 100 μg/ml ampicillin to OD_600_∼0.6–0.7. After induction with 0.5 mM IPTG the cells were further incubated O/N at room temperature and harvested by centrifugation at 5000 rpm, 4°C for 30 min. The cells were resuspended in cold MBP buffer (20 mM Tris-HCl (pH 7.4), 200 mM NaCl, 1 mM EDTA, 10 mM DTT) with 1x protease inhibitor. Lysozyme (Sigma Aldrich) was added to a final concentration of 1 mg/ml and the mixture was kept on ice for 20 min. The cells were lysed by tandem freezing (in liquid nitrogen until fully frozen) and thawing (at 37°C). A little-spatula tip of DNAase I was added to lysate and the mixture was kept on ice for 20 min. Freezing and thawing were repeated until the cloudy suspension became translucent. The extract was clarified by centrifugation (at 15000 rpm, 4°C for 20 min). The tST-MBP hybrid was purified from the crude cell extract using amylose resin affinity chromatography (New England BioLabs). The clarified extract (10 ml) was transferred to fresh amylose resin column (1 ml bead volume) and rocked gently at 4°C for 2 hr. Unbound material then were removed by centrifugation (at 2000 rpm, 4°C for 1 min). The resin was washed 3x with cold MBP buffer. The protein was eluted from resin by 2.5 ml elution buffer (MBP buffer, 10 mM matose).

### Gel analysis

**Figure 1:**
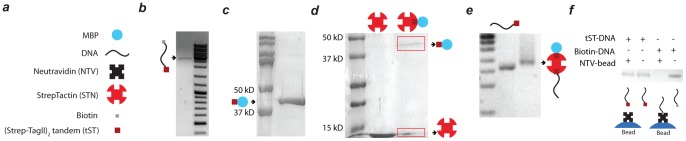
Hierarchical synthesis of protein-DNA hybrids. (a) Schematic drawing of the building blocks (b) 1% agarose gel demonstrating construction of tST-DNA-biotin hybrid at 2553 bps (c) SDS-PAGE analysis illustrating production of tST-MBP in *Ecoli* BL21.1 (d) SDS-PAGE characterization of STN-tST-MBP hybrid after amylose column purification. STN decomposes into monomers upon boiling. The schematic represents the expected dominant stoichiometry of the complex but does not exclude the possibility of minor amounts of complexes with other stoichiometries. (e) 1% agarose gel confirming the formation of multi protein-DNA hybrid (f) 1% agarose gel showing the presence and absence of DNA strand in the supernatant of incubated NTV beads by tST-DNA and biotin-DNA respectively. Biotinylated DNA easily binds to NTV, tST labelled DNA does not and remains in the supernatant.

DNA samples were analyzed by gel electrophoresis (Figure 1b, 1e and 1f) in non-denaturing 1% agarose gels in 0.5xTBE buffer at 80 V/cm. Agarose gels were stained with ethidium bromide (EtBr). Protein samples were applied on an 8% SDS-PAGE gel in 1x running buffer (190 mM Glycine, 25 mM Tris-base and 0.1% SDS) at 180 V/cm. SDS-PAGE gels were stained with Coomassie InstantBlue (Expedeon Ltd.).

### Bead preparation

Carboxylated polystyrene beads (Polysciences Inc.) were covalently linked to protein (STN, NTV, STV and AntiDig) via Carbodiimide reaction (PolyLink Protein Coupling Kit, Polysciences Inc.). Briefly, 25 μl of 1% (w/v) 1.87 μm diameter carboxylated polystyrene microspheres were washed twice by pelleting at 13.2 rpm (for 10 min) in a microcentrifuge tube and resuspending in coupling buffer (400 μl in first wash and 170 μl in second washing) (PolyLink Protein Coupling Kit, Polysciences Inc.). Then 20 μl of the freshly prepared EDCA solution (20 mg/ml; prepared by dissolving 1 mg EDCA in 50 μl coupling buffer) was added to the microparticle suspension and mixed gently end-over-end. After that 20 μg of desired protein (STN, NTV, STV and AntiDig) was added and mixture was incubated for 1 hr at room temperature with gentle mixing. The mixture then washed two times in 400 mμl storage buffer. Protein-coated beads were stored in 400 mμl storage buffer at 4°C until use.

DNA-coated microspheres were made by mixing ∼70 ng of dsDNA molecules and 1 μl protein-coated beads in 10 μl HMK (50 mM Hepes, pH 7.6, 100 mM KCl, 5 mM MgCl_2_) buffer. After 30 minutes incubation on a rotary mixer (4°C), the beads were diluted in 400 μl HMK buffer for use in optical tweezers experiments.

### Optical tweezers experiments

**Figure 2:**
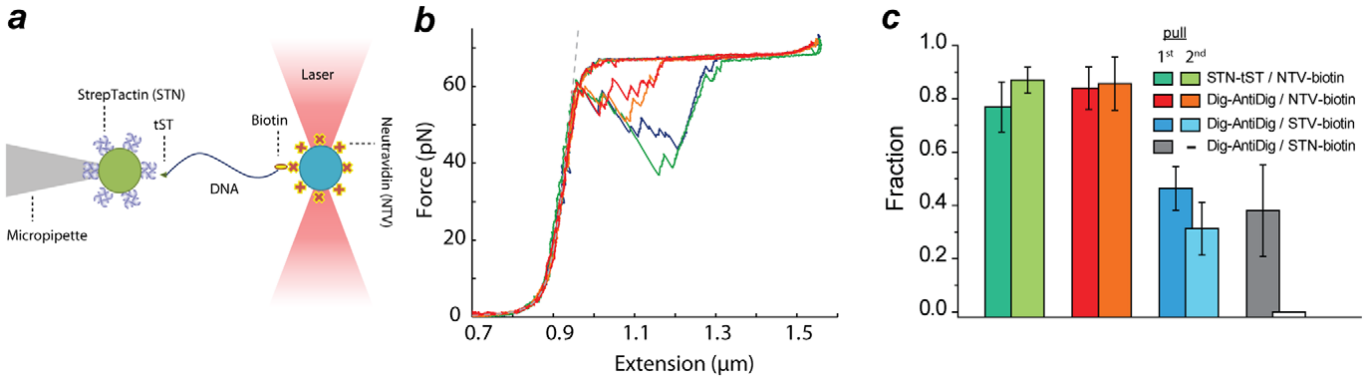
Mechanical stability analysis. (a) Optical tweezers setup (b) Force-extension curve of dsDNA showing overstretching at 65 pN, as well as the characteristic step-wise relaxation. The measured DNA stretching curves did not display additional steps that might have arisen from STN unfolding or its detachment from the surface. (c) Fraction of tethers that resisted 60 pN in first and second pull, compared between several commonly used linkage strategies and our proposed linkage strategies based on STN. For the (STN)biotin-DNA-Dig(AntiDig) system, almost all tethers broke at the first pull, and hence the subsequent pulls are not indicated.

The optical tweezers setup has been described elsewhere [13], [31]. Detection of forces on the trapped beadwas performed using back focal plane interferometry. Forces were recorded at 50 Hz. Trap stiffness and sensitivity were determined to be169±24 pN μm^−1^ and 2.74±0.24 V μm^−1^ respectively. A piezo-nanopositioning stage (Physik Instrumente) was used to move the sample cell and micropipette at a speed of 50 nm s^−1^. The beads were trapped in a flow chamber consisting of three parallel streams in laminar flow: one containing STN-coated beads; one containing NTV-coated beads with the DNA construct and a central buffer channel in which the measurements were conducted. Structure of the resulting molecular tether is schematically depicted in Figure 2a.

## Results and Discussion

### Polypeptide-DNA hybrids

To construct DNA molecules linked to polypeptide (tST-DNA), we used a primer covalently linked to the polypeptide. The PCR conditions were optimized to efficiently amplify the DNA from the template plasmid. By using gradient PCR, and testing several polymerases (Taq Polymerase and Phusion) and different PCR conditions, we found that comparatively long annealing and extension time (1 and 3 min per cycle respectively) allowed efficient amplification, resulting in a final yield of about 500 ng. The resulting construct was then characterized by agarose gel electrophoresis (Figure 1b) and later tested with an optical tweezers assay (Figure 2a).

### Polypeptide-protein hybrids

To synthesize a protein-polypeptide hybrid, we chose Maltose Binding Protein (MBP) as our model protein. MBP is a protein with a variety of applications in biotechnology and biological research, widely used to prototype a variety of biosensing platforms [32]. It is also a model protein for folding and export studies and is commonly used as fusion partner in protein biochemistry [13], [33].

The tST-MBP hybrid was constructed as described before. The hybrid was then tested with SDS-PAGE (Figure 1c), which showed a molecular weight between 37–50 kDa that corresponds well to the molecular weight of tST-MBP (∼42.5 kDa).

### Protein-DNA hybrids

Here we aimed to optimize the specific formation of a hybrid between MBP and DNA using ST-STN linkages (MBP-tST-STN-tST-DNA). Tetrameric structure of STN provides four binding sites for STs which in principle could allow for the formation of MBP-DNA complexes with different stoichiometries. It has been shown that for STV family, a 1∶1 stoichiometry can be successfully achieved by using excess amounts of ligand (e.g. biotin) or receptor (e.g. STV or AV) [34], [35].

To make this construct we first mixed STN (1 mg/ml) and tST-MBP (3 mg/ml) in 10∶1 ratio. Unbound STN was removed by amylose column purification. tST-MBP bound to amylose column was then eluted with maltose. SDS-PAGE (Figure 1d) showed two bands for eluted sample, with one corresponding to tST-MBP and one to STN only, thus showing that STN had successfully been bound to MBP. The previously constructed tST-DNA was then mixed with a large excess of the MBP-tST-STN hybrid (>30-fold molar excess) in order to favour binding of a single DNA molecule to each MBP. Agarose gel analysis showed a band distinctly above from tST-DNA, consistent with the formation of a MBP-tST-STN-tST-DNA hybrid (Figure 1e). As expected, MBP-tST-STN-tST-DNA hybrid shows a significantly reduced mobility as compared to tST-DNA due to its larger size and higher molecular weight. The successful formation of the complex hybrid also confirms the chemical structure of the constituting hybrids synthesized in the previous steps and the specificity of the linkages involved (Figure 1e and S1).

### Binding specificity

In many experiments, different specific linkages are typically required. For instance, when molecules are tethered between two beads in optical tweezers, each end is often attached with a different linkage. If the binding in these linkages would not be specific, both ends would bind to the same bead. Here we consider the two linkages tST-STN and biotin-NTV. To test whether NTV binds specifically to biotin and not to tST, NTV-coated beads were incubated either with tST-DNA or with biotin-DNA. After 30 min, beads were removed by centrifuging and supernatants were loaded onto an agarose gel (Figure 1f). The results showed that biotinylated DNA bound the beads efficiently, as no DNA could be detected in the supernatant. In contrast, all of the input tST-DNA remained in supernatant, showing no affinity to the beads. These results indicate that NTV binds selectively to biotin and not to tST, which is a central requirement for instance for efficiently tethering tST-DNA-biotin constructs between STN- and NTV-coated beads.

### Mechanical stability

To measure the mechanical stability of the linkage between tST and surface-bound STN, we pulled on a single synthesized DNA-tST-STN hybrid using optical tweezers. First, we immobilized tST-DNA-biotin constructs on NTV-coated beads by incubation for biotin-NTV linkage while keeping the tST-end free (Figure 2a). The NTV beads were titrated with varying amount of tST-DNA-biotin so that only few DNA constructs were linked to one bead. Next, the tST-STN linkage to beads coated with STN was established *in-situ*. Pulling curves showed overstretching at 65 pN, which indicated the presence of a single tether, and showed the tST-STN linkage was able to sustain such forces without breaking (Figure 2b). The measured DNA stretching curves did not display additional steps that might have arisen from STN unfolding or its detachment from surface.

Next, we performed a quantitative comparison of the mechanical stability of the tST-DNA-biotin and the biotin-DNA-Dig constructs. The latter is often used in optical tweezers studies in conjunction with STV- and AntiDig-coated beads [14], [20]. Note that in general, NTV-coated beads have advantages compared to STV-coated beads, given the higher affinity of NTV for biotin [23]. To compare the STN and Dig linkages, we performed pulling experiments on (NTV)biotin-DNA-Dig(Antidig) and (STN)tST-DNA-biotin(NTV) constructs, where the brackets indicate the two beads.

We considered a tether was established when the connections could sustain 20 pN. Connections that broke below 20 pN were disregarded (a maximum of 20% of tethers broke below 20 pN). The constructs were then stretched and relaxed multiple times with a displacement speed of 50 nm/sec to just beyond the DNA overstretching regime at about 65 pN, until the connection broke (N = 111 for the tST construct, N = 230 for the Dig constructs). We monitored the fraction of tethers able to sustain DNA overstretching, and distinguished first and subsequent pulls. Overall, we found quite similar results for the two constructs, with about 80% of the tethers able to sustain overstretching (Figure 2c). These data suggest that the tST-STN linkage has similar stability against applied force as incubated Dig-AntiDig in the first pull.

The stretching experiments indicated a number of additional points. For instance, for the tST-STN construct, subsequent pulls show a slight increase in the fraction of times the tether survives overstretching (Figure 2c, from 77% to 87%). A possible explanation for this increase could be the proposed bimodality of the ST-STN interaction [28]. The origin of this bimodality is believed to lie in the interaction of a single ST with a single or multiple sites on STN, where the latter is supposed to be somewhat less stable. Next, we performed additional experiments on (STV)biotin-DNA-Dig(AntiDig). These constructs showed an ability to sustain overstretching only in 40% of the cases, about half of what was found when using NTV and AntiDig beads. Thus, the biotin-STV linkage was significantly less stable than the biotin-NTV linkage, consistent with the significantly lower equilibrium binding constant for biotin-NTV [23].

When comparing all three bead-tether-bead constructs, additional observations can be made. First, the biotin-STV linkage makes the third of these constructs weaker than the first two. Thus, the biotin-STV linkage is less stable against applied force than the tST-STN linkage. The comparison also suggests that the biotin-STV linkage is less stable than the Dig-AntiDig linkage, as the latter contributes to the second construct that is very stable. This finding may be surprising, as biotin-STV is considered to be among the most stable linkages. To address this issue we hypothesised that the way in which the linkage is established could be important to stability in these experiments. Linkages can either form by incubation in bulk, during which there is a lot of time (order hour) and the molecules have many degrees of freedom. Linkages can also be formed *in-situ* within the tweezers apparatus by bringing the beads together, during which there is less time and fewer degrees of freedom. The former could yield more stable linkages than the latter.

To test this, we performed experiments where the Dig-AntiDig connection was formed *in-situ*, and contrasted this with earlier results where this connection was formed by bulk incubation. In this experiment, Dig-DNA-biotin molecules were incubated with NTV-coated beads, and the Dig-AntiDig connection was formed *in-situ* within the tweezers. Compared to the bulk-incubated Dig connection, the results indeed showed a significant reduction in the fraction of tethers that survived overstretching: a 34% reduction in the first pull and a 25% reduction in the second pull (Figure S2). To further investigate this issue we measured the time at which the tethers broke during sustained overstretching. For incubated Dig-AntiDig linkages, 7% of tethers broke in less than a second, while for in-situ established Dig-AntiDig linkages, 67% of tethers broke within that time (Figure S3). The same unbinding time has reported for fishing Dig-AntiDig connection where DNA molecules were bound to the STV-coated beads [36]. Thus, the Dig-AntiDig connection is significantly weaker when established *in-situ*. The type of AntiDig antibodies used may also affect stability. Polyclonal AntiDig antibodies are often used in single molecule pulling experiments [20], which could well bring significant variability in stability. The rupture force for a monoclonal AntiDig antibody was reported to be less than 20 pN for the pulling rate used in our study [37]. By incubation there may be a bias towards stronger Dig-AntiDig junctions. Importantly, in the experiments on the (STN)tST-DNA-biotin(NTV)construct (Fig. 2C), the tST-STN linkage was formed *in-situ*, showing that this linkage is not only stable but can also be formed rapidly.

Finally, a construct consisting of (STN)biotin-DNA-Dig(AntiDig) was able to sustain overstretching also in about 40% of the cases in the first pull. In these experiments, none of the tethers could sustain 65 pN in the second pull. The observed binding of STN to biotin does also illustrate the limitations of the specificity in this system: ST shows stable binding specifically to STN and not to NTV, but biotin binds stably both to STN and NTV, though more so to the latter. However, our protocol shows that these limitations can typically be overcome in practice, by first establishing the connection to biotin, which is less specific, and only then form the connection to tST which is specific.

**Figure 3:**
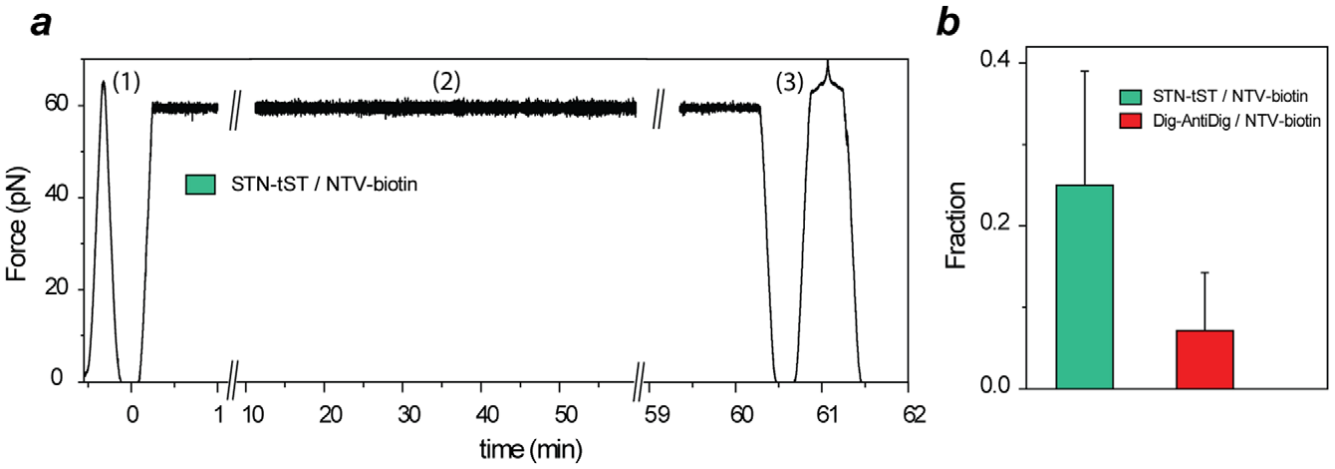
Mechanical stability analysis at constant force. (a) An example of stretched tether kept under a constant force of 60 pN in the second pulling cycle (2) by means of a force feedback for more than one hour. Stretching and relaxation cycles in the beginning (1) and at the end of the experiment (3) display a typical behaviour of dsDNA. (b) Fraction of the tethers resisting more than 10 min at 60 pN.

In order to probe the difference in stability between biotin-DNA-Dig and tST-DNA-biotin tethers more exhaustively, we tested for the ability to sustain high forces for long periods of time. In this experiment, tethers were first stretched to 65 pN and those that survived the first pull were kept under constant force of ∼ 60 pN until they broke. Figure 3a illustrates a case of (STN)tST-DNA-biotin(NTV) handle that could survive this load for an hour. In the second pull, the handle was stretched to 60 pN (in less than 1 min) and kept under force feedback for 60 min without breaking. Next, it was relaxed (Figure 3a, in between 60∶00 and 60∶30 min:sec) and showed a characteristic cycle of DNA overstretching (Figure 3a, in between 60∶30 and 61∶30 min:sec). The tether broke after additional 22 pulling cycles. Importantly, the fraction of strong tethers resisting more than 10 min at 60 pN in the second pull was found to be significantly higher with tST-STN as compared to Dig-AntiDig (Figure 3b). Thus, the tST-DNA-biotin handle is able to withstand high forces for longer than the biotin-DNA-Dig handle.

## Conclusions

We have presented a simple procedure to specifically attach a protein to a DNA molecule, using STN-tST linkages. The method is rapid and straightforward, and can be established *in-situ* within biologically relevant buffers. Binding of the DNA-tST construct to surface immobilized STN shows high mechanical stability, and can readily tolerate forces as high as 65 pN for tens of minutes. The engineered linkage can be used as a reliable linker for optical tweezers studies of proteins and nucleic acids, both in constant pulling rate and force modes [38]–[40].

The motivation to use STN to end-join two molecules was based on reported high rupture forces (40 pN and 60 pN) [28]. We found that the average rupture force was beyond the overstretching transition of 65 pN for the ST-STN linkage studied here, which may be due to the dual ST repeats or other experimental differences. The specificity, stability, and rapid *in-situ* formation of the STN-tST complex allows it to be used in combination with other well-used linkages that can also be stably formed *in-situ*, such as NTV-biotin. Dig-AntiDig linkages of similar stability can be formed, but they require bulk incubation. Thus, choice of linkage depends on the precise application and formation possibilities. We find that tST-STN is more stable against applied force than the commonly used biotin-STV linkage. Moreover, we show that tST-STN can be used for surface attachments as well as for linkage between DNA and protein molecules, which has not been achieved for Dig-AntiDig linkages. Because of the high stability of STN, this complex could potentially also be used in a broad thermal range and harsh conditions.

We have shown that constructing tST-DNA hybrids is straightforward using PCR amplification, making our method suitable for broad applications. For single molecule studies, the presented approach could be applied in combination with other peptide-DNA hybrids. For example, halo tags-DNA hybrid could be constructed as a handle and be linked covalently to halogenase-coated beads. Similarly, a peptide substrate to ubiquitin ligase could be used to generate peptide-DNA hybrid and then be linked to the protein ligase-coated bead. The reversibility of the ST-STN reaction, using Desthiobiotin [24], will make the ST-STN linkage also highly suitable for biologically inspired soft matter systems, where reversibility could open up new possibilities.

## Supporting Information

Figure S1 **The specificity of tST-STN interactions.** (a) 1% Agarose gel showing protein-DNA hybrid (shown in Figure 1e) does not form in the absence of ST. Unlabeled DNA (25 ng) was mixed with a large excess of unlabeled MBP (3 μg) and STN (1 μg). The mixtures were incubated for 1 hour in 4°C and then loaded in to the 1% agarose gel. In contrast to tST-DNA, unlabeled DNA does not bind STN. In lane 2, the band appears exactly where DNA band appears in lane 1, indicating that DNA and STN do not form a complex. A gel analysis on the mixture of tST-MBP and DNA also results in a band at the same location as DNA alone. This experiment confirms that for the formation of the hybrid shown in Figure 1e, specific tST-STN interactions are required. (b) SDS-PAGE analysis illustrates that STN does not bind to MBP in the absence of ST (Experiment A) and cannot be eluted from amylose resin by maltose (Experiment B). Unlabeled MBP (0.15 mgr) was added to the STN (0.25 mgr) and the mixture was incubated for 1 hour in 4°C (Experiment A). The complex was then combined with amylose resin (for 2 hour in 4°C) and the resin subsequently was washed with maltose. SDS-PAGE showed STN band in master mixture (MBP+STN) (1) and supernatant sample (2), but no band was detected for eluted sample at the same location (3). This confirms that MBP-STN complex does not form specifically, in the absence of ST linkage. This process was repeated with the pure STN solution (Experiment B). The result shows that STN molecules which bind the column nonspecifically cannot be eluted by maltose. Overall, these control experiments indicate that the eluted STN molecules in Figure 1d, were linked via tST to MBP and confirm the chemical structure of the synthesized MBP-tST-STN complex.
(TIF)

Figure S2 **Fraction of (NTV)biotin-DNA-Dig(AntiDig) tethers that resisted 60 pN in first and second pull, compared between different methods of Dig-AntiDig establishment.** Connections can either form by incubation in bulk or *in-situ* within the tweezers apparatus by bringing the beads together. In blue bars, Dig-DNA-biotin molecules were incubated with NTV-coated beads, and the Dig-AntiDig connection was formed *in-situ*. In purple bars, Dig-DNA-biotin molecules were incubated with AntiDig-coated beads, and the biotin-NTV connection was formed *in-situ*. The statistics show a reduction in the fraction of survived tethers (both in the first and second pull) when Dig-AntiDig linkage formed *in-situ*.
(TIF)

Figure S3 **Histogram of unbinding time of a tethered (NTV)biotin-DNA-Dig(AntiDig) held at overstretching, compared between different methods of Dig-AntiDig establishment.** Linkages can either form by incubation in bulk or *in-situ* within the tweezers. In blue bars, Dig-DNA-biotin molecules were incubated with NTV-coated beads, and the Dig-AntiDig connection was formed *in-situ* within the tweezers. In purple bars, Dig-DNA-biotin molecules were incubated with AntiDig-coated beads, and the biotin-NTV connection was formed *in-situ*. The statistics show most of the *in-situ* formed Dig-AntiDig connections broke immediately (blue bars), while a few number of Dig-AntiDig linkages which formed by incubation (purple bars), broke within that time.
(TIF)
