# Supplementary figures and images for: A Polypeptide-DNA Hybrid with Selective Linking Capability Applied to Single Molecule Nano-Mechanical Measurements Using Optical Tweezers
Source: PLoS One. 2013 Jan 15;8(1):e54440. doi: 10.1371/journal.pone.0054440 (PMC3545873; doi:10.1371/journal.pone.0054440)

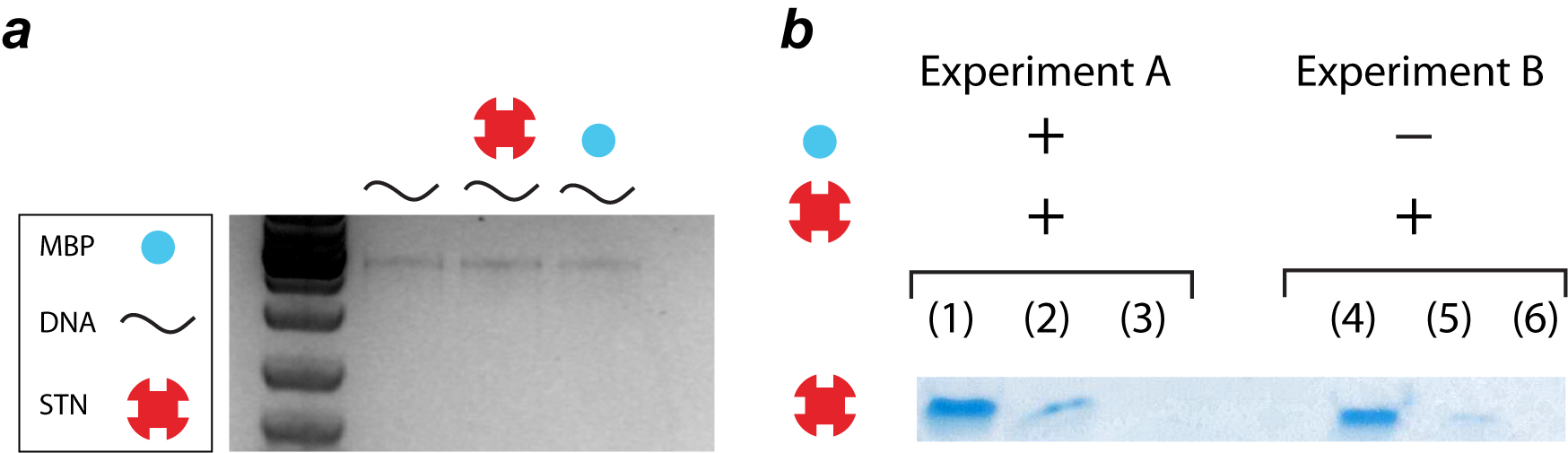

Supplement: Figure S1 — The specificity of tST-STN interactions. (a) 1% Agarose gel showing protein-DNA hybrid (shown in Figure 1e) does not form in the absence of ST. Unlabeled DNA (25 ng) was mixed with a large excess of unlabeled MBP (3 μg) and STN (1 μg). The mixtures were incubated for 1 hour in 4°C and then loaded in to the 1% agarose gel. In contrast to tST-DNA, unlabeled DNA does not bind STN. In lane 2, the band appears exactly where DNA band appears in lane 1, indicating that DNA and STN do not form a complex. A gel analysis on the mixture of tST-MBP and DNA also results in a band at the same location as DNA alone. This experiment confirms that for the formation of the hybrid shown in Figure 1e, specific tST-STN interactions are required. (b) SDS-PAGE analysis illustrates that STN does not bind to MBP in the absence of ST (Experiment A) and cannot be eluted from amylose resin by maltose (Experiment B). Unlabeled MBP (0.15 mgr) was added to the STN (0.25 mgr) and the mixture was incubated for 1 hour in 4°C (Experiment A). The complex was then combined with amylose resin (for 2 hour in 4°C) and the resin subsequently was washed with maltose. SDS-PAGE showed STN band in master mixture (MBP+STN) (1) and supernatant sample (2), but no band was detected for eluted sample at the same location (3). This confirms that MBP-STN complex does not form specifically, in the absence of ST linkage. This process was repeated with the pure STN solution (Experiment B). The result shows that STN molecules which bind the column nonspecifically cannot be eluted by maltose. Overall, these control experiments indicate that the eluted STN molecules in Figure 1d, were linked via tST to MBP and confirm the chemical structure of the synthesized MBP-tST-STN complex. (TIF) [file pone.0054440.s001.tif]

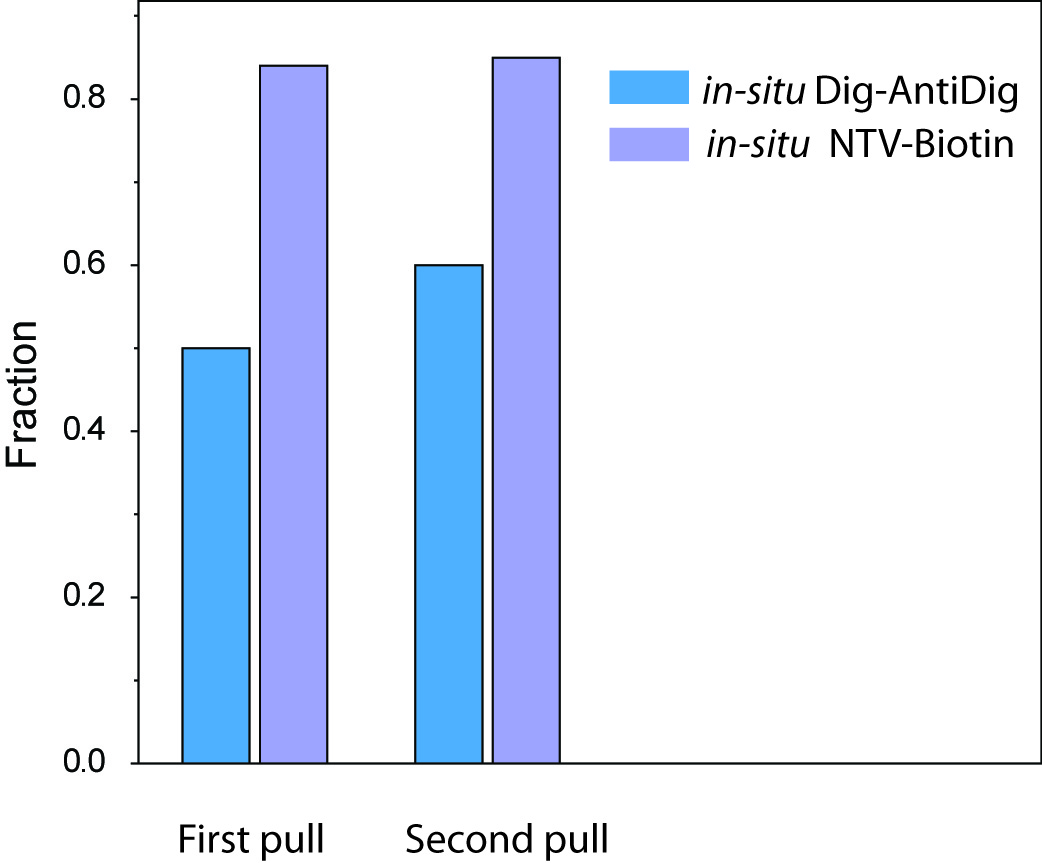

Supplement: Figure S2 — Fraction of (NTV)biotin-DNA-Dig(AntiDig) tethers that resisted 60 pN in first and second pull, compared between different methods of Dig-AntiDig establishment. Connections can either form by incubation in bulk or in-situ within the tweezers apparatus by bringing the beads together. In blue bars, Dig-DNA-biotin molecules were incubated with NTV-coated beads, and the Dig-AntiDig connection was formed in-situ. In purple bars, Dig-DNA-biotin molecules were incubated with AntiDig-coated beads, and the biotin-NTV connection was formed in-situ. The statistics show a reduction in the fraction of survived tethers (both in the first and second pull) when Dig-AntiDig linkage formed in-situ. (TIF) [file pone.0054440.s002.tif]

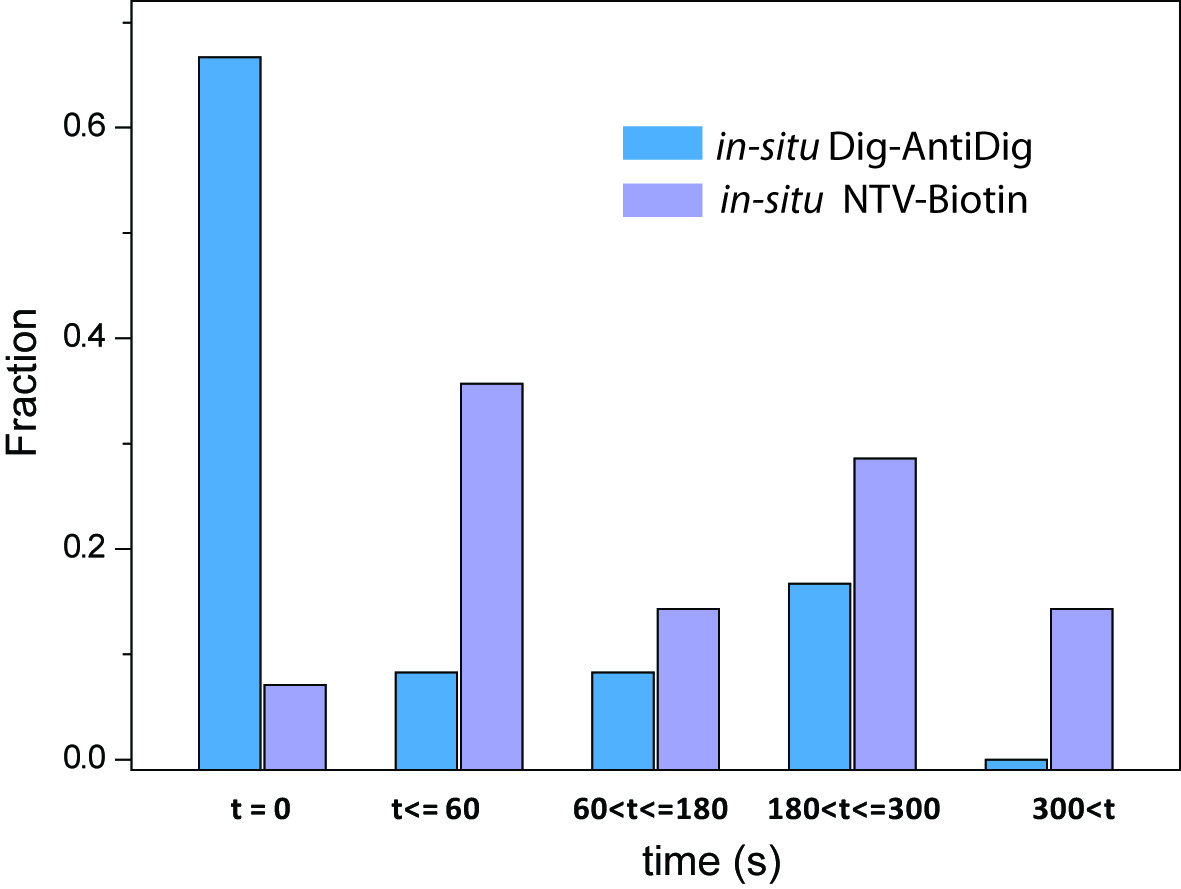

Supplement: Figure S3 — Histogram of unbinding time of a tethered (NTV)biotin-DNA-Dig(AntiDig) held at overstretching, compared between different methods of Dig-AntiDig establishment. Linkages can either form by incubation in bulk or in-situ within the tweezers. In blue bars, Dig-DNA-biotin molecules were incubated with NTV-coated beads, and the Dig-AntiDig connection was formed in-situ within the tweezers. In purple bars, Dig-DNA-biotin molecules were incubated with AntiDig-coated beads, and the biotin-NTV connection was formed in-situ. The statistics show most of the in-situ formed Dig-AntiDig connections broke immediately (blue bars), while a few number of Dig-AntiDig linkages which formed by incubation (purple bars), broke within that time. (TIF) [file pone.0054440.s003.tif]
